# Defective lysosomal acidification: a new prognostic marker and therapeutic target for neurodegenerative diseases

**DOI:** 10.1186/s40035-023-00362-0

**Published:** 2023-06-08

**Authors:** Chih Hung Lo, Jialiu Zeng

**Affiliations:** Lee Kong Chian School of Medicine, Nanyang Technological University, Singapore, 308232 Singapore

**Keywords:** Neurodegenerative diseases, Alzheimer’s disease, Parkinson’s disease, Lysosomal de-acidification, Autophagy dysfunction, Early detection, Prognostic marker, Small molecules, Nanomedicine, Nanoparticles

## Abstract

Lysosomal acidification dysfunction has been implicated as a key driving factor in the pathogenesis of neurodegenerative diseases, including Alzheimer’s disease and Parkinson’s disease. Multiple genetic factors have been linked to lysosomal de-acidification through impairing the vacuolar-type ATPase and ion channels on the organelle membrane. Similar lysosomal abnormalities are also present in sporadic forms of neurodegeneration, although the underlying pathogenic mechanisms are unclear and remain to be investigated. Importantly, recent studies have revealed early occurrence of lysosomal acidification impairment before the onset of neurodegeneration and late-stage pathology. However, there is a lack of methods for organelle pH monitoring in vivo and a dearth of lysosome-acidifying therapeutic agents. Here, we summarize and present evidence for the notion of defective lysosomal acidification as an early indicator of neurodegeneration and urge the critical need for technological advancement in developing tools for lysosomal pH monitoring and detection both in vivo and for clinical applications. We further discuss current preclinical pharmacological agents that modulate lysosomal acidification, including small molecules and nanomedicine, and their potential clinical translation into lysosome-targeting therapies. Both timely detection of lysosomal dysfunction and development of therapeutics that restore lysosomal function represent paradigm shifts in targeting neurodegenerative diseases.

## Introduction

**Fig. 1:**
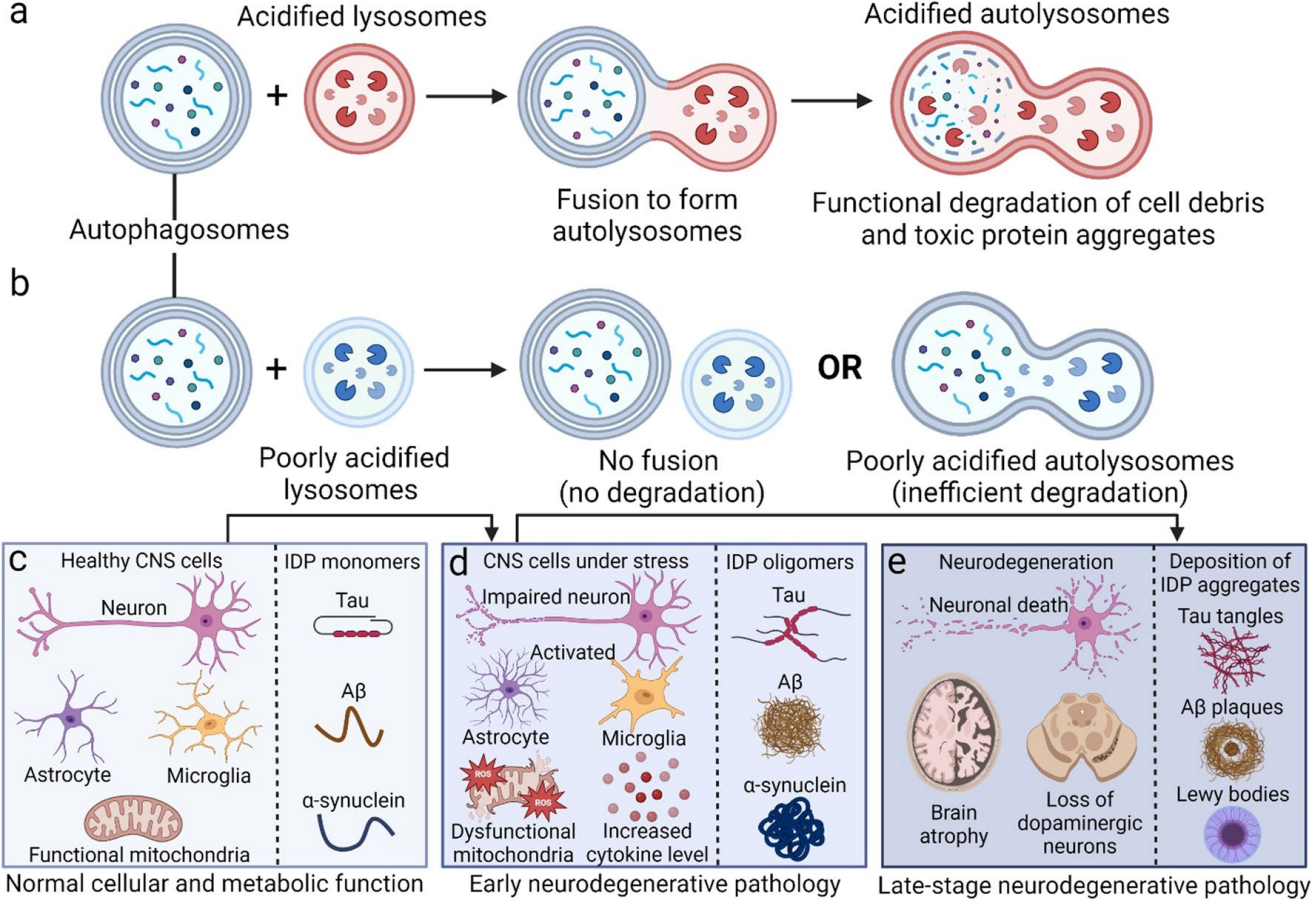
Role of lysosomal acidification dysfunction in early neurodegenerative pathology. **a** Under normal physiological conditions, acidified lysosomes fuse with autophagosomes to form sufficiently acidified autolysosomes which can efficiently degrade accumulated cell debris and toxic protein aggregates. **b** Under pathological conditions, lysosomes with elevated pH either have no fusion with autophagosomes leading to no degradation, or fuse with autophagosomes to form poorly acidified autolysosomes which are inefficient in cellular degradation. **c** Poorly acidified lysosomes induce alterations from normal cellular and metabolic functions to early neurodegenerative pathology and could act as an early indicator of disease pathogenesis. **d** Pathogenesis of neurodegenerative diseases initiates with early pathology including increases of neuroinflammatory cytokines, activated glial cells, impaired neurotransmission, mitochondrial dysfunction, reactive oxygen species (ROS) production, and accumulation of pre-fibrillar, oligomeric toxic intrinsically disordered protein (IDP) aggregates due to inefficient cellular degradation by poorly acidified autolysosomes. **e** Late-stage neurodegeneration pathology includes the presence of toxic protein aggregates such as tau neurofibrillary tangles, Aβ plaques, and Lewy bodies with α-synuclein, as well as neuronal death. Schematics were created by BioRender.

Lysosomal dysfunction in neurons and glial cells contributes to neurodegenerative diseases including Alzheimer’s disease (AD), frontotemporal dementia (FTD), Parkinson’s disease (PD), and amyotrophic lateral sclerosis (ALS), and has been a major field of research in the past decades [1, 2]. Lysosome functions as a major signaling hub and regulatory platform for cellular processes and controls cell death and survival [3, 4]. As the endpoint of autophagic and phagocytic networks, lysosome serves as a central mediator to ensure the completion of the cellular degradation process. An optimal lysosomal acidification of pH 4.0–5.0 is essential for fusion with autophagosomes and maintenance of normal autolysosomal functions, including enzyme activity, organelle biogenesis, and effective clearance of unwanted cellular components, cell debris, and accumulated toxic protein aggregates (Fig. 1a) [5, 6].

Lysosomal acidification is maintained primarily by a proton-pump known as vacuolar (H+)-ATPase (V-ATPase), together with multiple ion channels found on the lysosomal membrane such as the transient receptor potential (TRP) channels, two-pore channels (TPC), transmembrane protein 175 (TMEM175), and chloride-proton exchanger that facilitates the exchange of ions including Ca^2+^, Na^+^, K^+^, and Cl^−^, where they play a synergistic role in regulating lysosomal acidification [5]. Importantly, V-ATPase is a multimeric enzyme complex that pumps protons from the cytosol into the lysosomal lumen, among which the most important subunit is  the V1 domain in association with the membrane-bound V0 sector [5]. Defective lysosomal acidification in the central nervous system (CNS) leads to inefficient cellular degradation by poorly acidified autolysosomes (Fig. 1b) and has been associated with early neurodegenerative pathology including the accumulation of toxic protein aggregates (Fig. 1c, d), prior to late-stage pathology of neuronal death and toxic protein deposition (Fig. 1e).

AD is the most prevalent neurodegenerative disease, where abnormalities of the autolysosomal system are a distinct neuropathological feature [7]. Multiple gene mutations associated with familial AD (FAD) have been reported to affect lysosomal acidification [7–9], with autosomal dominant mutations of *PSEN1* (presenilin-1, PS1) [10–12] and *APP* (amyloid precursor protein, APP) [13, 14] being prominent examples. Blastocysts from *PSEN1-*knockout mice exhibit reduced expression of V-ATPase subunit V0a1 as well as defective maturation, trafficking, and assembly of the V-ATPase enzyme complex, resulting in lysosomal acidification dysfunction [10, 11]. However, another study reported no significant difference in lysosomal pH in embryonic stem cells lacking *PSEN1* or both *PSEN1* and *PSEN2* as compared to the control [15]. In other studies, Tyr^682^-phosphorylated APP β-C-terminal fragment and the internalized β-amyloid (Aβ) have been shown to interact with the cytosolic V0a1 subunit and luminal V0C subunit of V-ATPase, respectively, thereby disrupting the activity of the enzyme complex [14]. Recent studies have demonstrated in different AD mouse models with *APP* and *PSEN1*  mutations, that early lysosomal acidification defects reduce autophagic degradation and increase accumulation of Aβ in neurons and late-stage deposition of Aβ plaques [12].

In FTD and ALS, mutations of several genes including *PGRN*, *TMEM106B*, *VCP*, *TARDBP*, *UBQLN2*, and *MAPT*, together with their pathogenic protein functions, have been implicated in lysosomal acidification dysfunction and autophagy impairment [16, 17]. Specifically, in neurons with *MAPT* P301S mutation derived from genetically engineered induced pluripotent stem cells (iPSCs), reduced lysosomal acidification is observed prior to the onset of tau oligomerization and the development of neurodegeneration [18]. It has also been suggested that phospho-tau interacts with the cytosolic V1B2 subunit of V-ATPase to inhibit  the activity of the latter [19]. Other studies have illustrated impairments in autophagic flux and autolysosomal functions, suggesting the presence of lysosomal acidification defects in several mouse models of tauopathy [19–22].

Similarly, in PD, several genes have been reported to be directly linked to impaired lysosomal acidification and function [22], including *LRRK2*, *ATP6AP2*, *ATP13A2*, *ATP10B*, and *SNCA* [7]. While *LRRK2* and *ATP6AP2* mutations are associated with reduction of V-ATPase activity by altering the trafficking and assembly of the enzyme complex [23, 24], *ATP13A2* (P5-type ATPase) [25] and *ATP10B* (P4-type ATPase) [26] mutations cause the accumulation of polyamines and lipids, respectively [25, 26], resulting in elevated lysosomal pH in neurons. The overexpression of mutant α-synuclein, including A53T and A30P, have also been shown to lead to lysosomal de-acidification in SH-SY5Y cells [27]. Interestingly, the deficiency of *TMEM175*, which encodes for a K^+^ channel, induces lysosomal over-acidification, instead of de-acidification, but similarly causes impaired cathepsin activity and α-synuclein aggregation both in vitro and in vivo [28, 29]. Moreover, other environmental factors that contribute to sporadic models of PD, including neurotoxins 1-methyl-4-phenyl-1,2,3,6-tetrahydropyridine (MPTP) [30, 31] and 6-hydroxidopamine [31], as well as human Lewy bodies containing α-synuclein [32], have been shown to elevate lysosomal pH in both cellular and mouse models.

The rapidly growing numbers of identified gene mutations and environmental factors highlight the pathogenic importance of lysosomal acidification dysregulation in neurodegenerative diseases. This is further exemplified by the association of lysosomal storage diseases, which are characterized by defective lysosomal enzymes and abnormal accumulation of undegraded substrates inside lysosomes, with progressive neurodegenerative phenotypes and early death [33–35]. While recent studies have provided clear evidence that defective lysosomal acidification develops before pathological manifestations in AD, there is a lack of direct evidence for early lysosomal abnormalities in PD, mainly due to the lack of PD mouse models with in vivo autophagic flux reporter that reflects the extent of lysosomal acidification. Despite this, multiple PD studies have shown autolysosomal impairments and demonstrated effectiveness of lysosome-targeting therapeutic agents for restoration of luminal acidification to rescue neurodegenerative pathology, suggesting lysosomal acidification dysfunction in PD. Here, we consolidate evidence, both direct and indirect, that supports impaired lysosomal acidification as a primary driver and an early indicator of neurodegeneration. We propose that early detection of lysosomal acidification dysfunction would allow for more accurate identification of potential pathological manifestations and enable timely treatment.

## Early indicator of neurodegeneration

One of the important discoveries in yeast models is that the maintenance of optimal pH of lysosome-like vacuole positively regulates mitochondrial function and delays the aging process [36]. The study also revealed that the vacuolar acidity decline occurs during the early asymmetric divisions of a mother cell, preceding mitochondrial dysfunction and leading to reduced number of cell divisions or shortened lifespans [36]. In *Drosophila*, early loss of the V0a1 subunit in photoreceptor neurons leads to defective lysosomal acidification in larval eye discs, increases their vulnerability to neurotoxic Aβ and tau proteins, and contributes to adult-onset progressive degeneration [37]. Conditional knockdown or deletion of *ATP6AP2* (encodes for ATP6AP2, an essential accessory component of V-ATPase), leads to the appearance of autophagic vacuoles, and subsequent neurodegeneration and cognitive impairment in both *Drosophila* and mouse models [38]. This is supported by variants of  *ATP6AP2* which decrease the protein level and compromise V-ATPase activity in cells [39]. These results suggest that partial reduction in lysosomal acidity does not immediately inhibit the autophagic degradative processes but renders the system more susceptible to failure over time.

In AD, a recent landmark study monitored autophagy flux and lysosomal acidification in five AD mouse models using a neuron-specific transgenic mRFP-eGFP-LC3 probe for autophagy and lysosomal pH monitoring. Findings show that early lysosomal acidification dysfunction occurs well before neurodegeneration. The mice expressing wild-type (WT) human APP or mutant human APP or a combination of mutant human APP and PS1 consistently show early faulty lysosomal acidification characterized by the PANTHOS (flower-shaped blebs) pattern, which was detectable as early as 5–6 months before extracellular Aβ deposition [12]. Similar PANTHOS phenomena driving neuronal death, including lysosome enlargement and membrane permeabilization, cathepsin release, glial activation, and Aβ plaque formation, were also present in post-mortem AD brain tissues at the Braak II stage, suggesting early lysosomal acidification defects in sporadic AD [12, 40]. This is further supported by another study showing that lysosome deficiency is an early pathogenic event in NHE6 (Na+/H+  exchanger 6)-null rat brain, preceding autophagic dysfunction and loss of pyramidal cells, resulting in aggregation of Aβ and tau and neurodegeneration [41].

In tauopathies, an elevation of lysosomal pH was observed in iPSC-derived neurons with IVS10 + 16/P301S mutations of *MAPT* at day in vitro (DIV) 65 compared with IVS10 + 16/WT control neurons. Upon elevation of lysosomal pH, there was increased phosphorylation of tau at Ser396/Ser404 (PHF1) and Thr181 (AT270) in IVS10 + 16/P301S neurons at DIV80, suggesting that early defects in lysosomal acidification could contribute to increased tau pathology and subsequent neurodegeneration [18]. In other studies, phospho-tau has been shown to interact with the ATP6V1B2 subunit of the V-ATPase [19, 42]. Importantly, ATP6V1B2 subunit is known to have a reduced expression in early development of AD pathology [43], disrupt V-ATPase activity, impair lysosomal function, and lead to neurodegeneration. This is further supported by studies reporting that autophagic and proteasomal impairments occur before tau aggregation [44] as well as the observation of autophagic and lysosomal defects in post-mortem human brain tissues with tauopathies (early onset of FAD) [45]. As some of the AD mouse models contain multiple protein variants, it is important to dissect the contribution of each protein and the associated mutations to AD pathogenesis.

In PD, lysosomal acidification impairment contributes to mitochondrial dysfunction, reduced clearance of α-synuclein, Lewy body pathology and neurodegeneration [46]. While multiple genes have been associated with lysosomal acidification dysfunction in PD [47], the early occurrence of this defect in the course of the disease has not been clearly delineated, although indirect evidence has been shown by several studies. Loss-of-function mutations in *ATP13A2* impair lysosomal acidification and are associated with early-onset parkinsonism [46, 48, 49]. Although alterations in lysosomal pH were not monitored, *ATP13A2*^−/−^ mice showed age-dependent autophagy impairment at 10 to 18 months of age [50], but displayed late-onset sensorimotor deficits at 20 to 29 months of age [51]. The expression of *LRRK2*-R1441C has been shown to elevate lysosomal pH and induce a decrease in autophagosome/lysosome fusion in primary cortical neuronal cultures [24]. Similar lysosomal abnormalities have been observed in *LRRK2*-R1441C mice at 22 months [24], but motor deficits are only observed in mice older than 24 months [52]. In a previous study, mice were injected with MPTP for five consecutive days and sacrificed at different time-points after the last MPTP injection. Autophagic and lysosomal dysfunction were observed on day 1, preceding dopaminergic neuronal death observed on days 2 to 4, suggesting the role of early autolysosomal dysfunction in PD pathogenesis [53].

In sum, evidence from both AD and PD studies suggests that dysfunctional V-ATPase and defective lysosomal acidification are critical triggering mechanisms of the pathological features of neurodegeneration. Therefore, strategies for monitoring the extent of lysosomal acidification in vivo are needed for effective disease prognosis and management.

## Prognostic markers and diagnostic tools associated with lysosomal acidification dysfunction

**Fig. 2:**
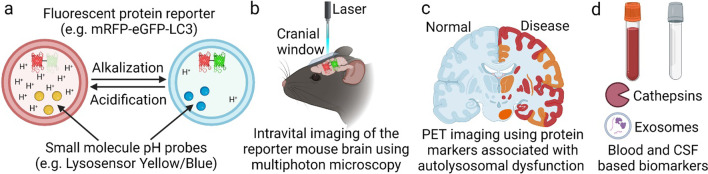
Strategies and methods for detecting autolysosomal dysfunction. **a** Detection of changes in autolysosomal acidification using fluorescent protein reporter (e.g., mRFP-eGFP-LC3) and small-molecule pH probes (e.g., Lysosensor Yellow/Blue). **b** Monitoring of autolysosomal acidification in vivo through intravital imaging of the reporter mouse brain using multiphoton microscopy. **c** Positron emission tomography (PET) imaging using protein markers that correlate with autolysosomal dysfunction to detect early onset of neurodegeneration in patients. **d** Blood- and CSF-based biomarkers that are associated with autolysosomal dysfunction, such as cathepsin level/activity and other molecular signatures obtained from CNS-derived exosomes, are potential early indicators of neurodegeneration. Schematics were created by BioRender.

Early detection of lysosomal acidification alterations and identification of protein markers for dysregulation of autolysosomal network and pathways would aid in the prognosis of neurodegenerative diseases. The extent of lysosomal acidification is typically measured by using fluorescent probes (e.g., LysoTracker™ or Lysosensor™ Yellow/Blue) [54] or reporter plasmids (e.g., mRFP-eGFP-LC3 or mCherry-LAMP1-mTFP1 (FIRE-pHLy)) [12, 55] where the fluorescence changes depending on the pH of the local environment (Fig. 2a). These techniques are mostly applicable to studies in living cells. Recently, the Wang group has developed assays to examine lysosomal acidification and cleavage activity in vivo, by using *Caenorhabditis elegans* expressing fluorescent reporters as a model system [56]. Importantly, *C. elegans* showed declines of lysosomal acidity and degradation activity during aging and under stressed conditions, making them a suitable tool to study longevity and neurodegenerative pathways [57]. In another study, the Nixon group developed an autophagy reporter mouse expressing mRFP-eGFP-LC3 in neurons driven by the Thy1 promoter, to directly measure alterations of lysosomal acidification and autophagic flux in vivo [58]. Brain tissues of AD mouse models expressing this reporter have been observed to reflect lysosomal acidification impairments and neurodegenerative pathology [12]. This critical study opens avenue for real-time in vivo monitoring of lysosomal acidification in brains of living mice, which may potentially be achieved with a cranial window set-up and multiphoton microscopy to elucidate early-stage pathogenic mechanisms of AD (Fig. 2b), although this would be an invasive procedure.

The translation of lysosomal acidification and related functions as early indicators of neurodegeneration in humans would be a major step forward. Initial studies, although not yet done in CNS cells, have confirmed an elevation of lysosomal pH by 0.2–0.3 units and 0.6 units in primary dermal fibroblasts with PS1 A246E mutation obtained from patients with FAD [59] and in fibroblasts from patients with Down syndrome [13], respectively. To step further, it is theoretically possible to measure lysosomal acidification and function in the CNS by positron emission tomography (PET) imaging with probes that target lysosome contents [60] or protein markers for lysosomal dysfunction (Fig. 2c) [61]. There is also a need for new designs of PET probes that can cross the blood-brain barrier (BBB) and target lysosomes, providing signals that differ in acidified vs. de-acidified environments [62].

Blood and cerebrospinal fluid (CSF) biomarkers for lysosomal dysfunction have been studied for early detection and diagnosis of neurodegenerative diseases (Fig. 2d). Among all the protein markers studied, cathepsins such as cathepsins B, D, E, and F are most closely associated with the extent of lysosomal acidification, as their activities and localization within lysosomes or translocation into the cytosol and extracellular space [63]  is highly dependent on lysosomal pH [6]. While there are inconsistent results, there is an overall trend illustrating an increase of cathepsin levels in biofluids and tissues of AD and a decrease in PD. However, the functional activities of cathepsins remain to be clarified [64]. The increase of cathepsin level in AD is consistent with the observation that lysosomal alkalization leads to increased mRNA levels of cathepsins and lysosomal permeabilization, resulting in increased cathepsin levels in the cytosol and the extracellular space [6, 65–68]. Decreased cathepsin levels in blood and post-mortem tissues of PD patients are potentially due to cathepsin release through exosomes or exocytosis [69, 70]. More detailed and rigorous analyses of patient samples with more consistent and reliable results are needed to advance the measurement of cathepsin levels and activities as diagnostic tools for AD and PD. There are also studies suggesting the measurements of lysosomal acidification and function in immune cells from blood or CSF as indicators of immune activation and inflammation [71], although this needs to be verified by more evidence from studies associated with neurodegenerative diseases.

CNS-derived exosomes in blood or CSF, which contain brain-specific cargos, are emerging biomarkers as they can be isolated by minimally invasive methods and their molecular profiles closely reflect the biochemical alterations in the CNS [72]. Being regarded as the mini-version of parental cells [73], the CNS-derived exosomes are theoretically capable of reflecting the status of lysosomal acidification and function in neurons and glial cells in the brain. An initial step would be to re-acidify lysosomes impaired under disease conditions and compare the omics (e.g., transcriptomic, proteomic, metabolomic, and lipidomic) signatures of the CNS-derived exosomes between the diseased and the recovered conditions. The differentially expressed genes or proteins in the exosomes may reveal mechanistic links between defective lysosomal acidification and the pathogenesis of neurodegenerative diseases and identify new molecular targets for therapeutic intervention. To work towards clinical applications of exosome-based biomarkers, reliable protocols of vesicle isolation, marker detection, and data analysis should be established to ensure robustness and high reproducibility.

## Restoring lysosomal acidification as a therapeutic intervention

**Fig. 3:**
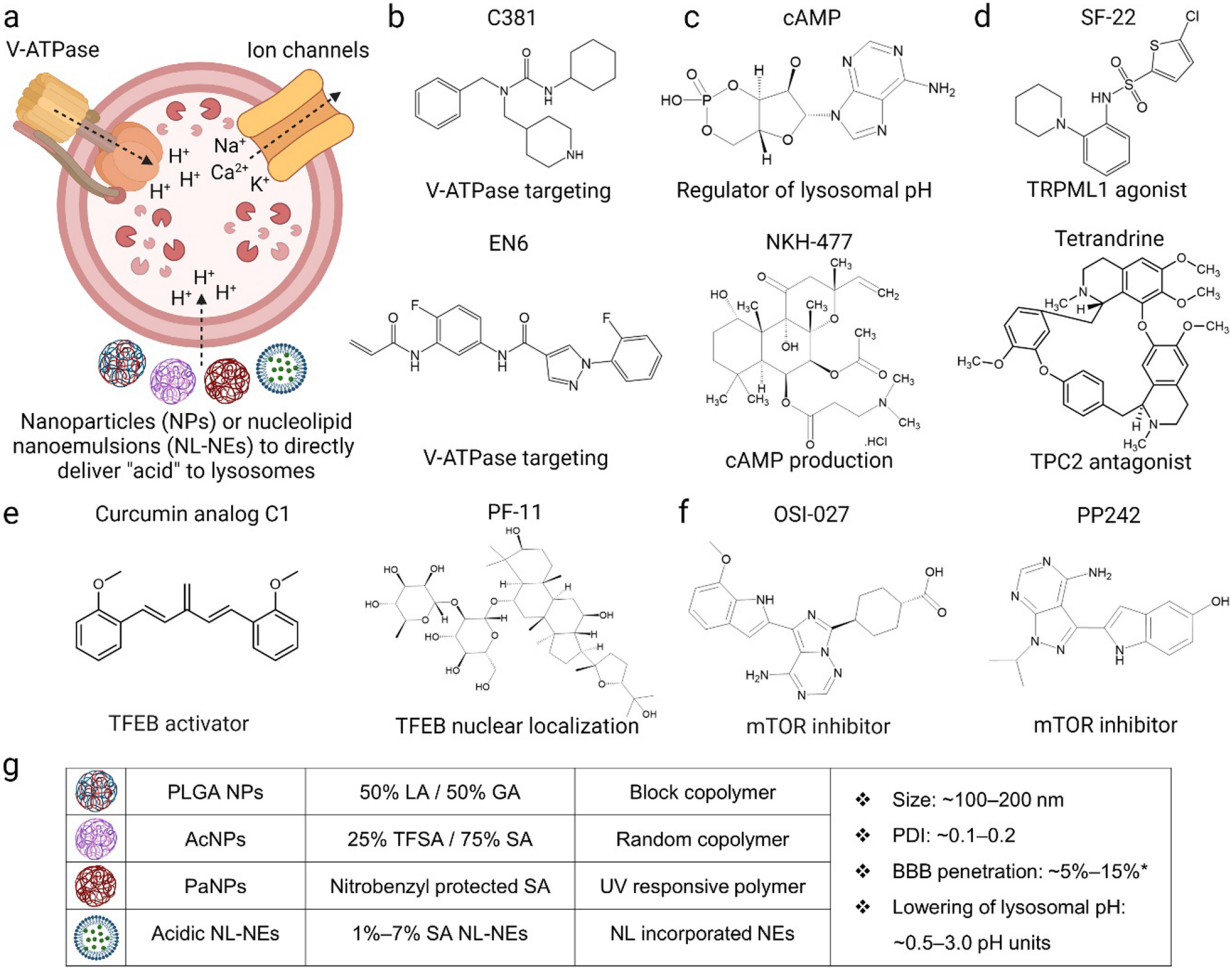
Lysosomal acidification-modulating agents. **a** Schematic representation of lysosomal acidification modulation by small molecules and lysosome-targeting  nanomedicine. **b** C381 and EN6 activate V-ATPase to promote the proton-pumping capacity of the enzyme complex. **c** Cyclic adenosine monophosphate (cAMP, regulator of lysosomal pH) and NKH-477 (adenylyl cyclase activator) increase the intracellular content of cAMP, which is essential for maintaining lysosomal acidification. **d** Targeting ion channels, such as activating TRPML1 by SF-22 and inhibiting TPC2 by tetrandrine, allows for a balance of ions in the lysosome lumen to maintain lysosomal acidification. **e** Curcumin analog C1 and PF11 activate transcription factor EB (TFEB) and increase its nuclear localization to enable the expression of lysosomal components and enhance luminal acidification. **f** OSI-027 and PP242 acidify lysosomes by inhibiting mTOR signaling. **g** Lysosome-targeting  nanoparticles (NPs) or nucleolipid nanoemulsions (NL-NEs) formed by various chemical compositions. Compositions of the NPs or NL-NEs shown are based on the most widely used formulations that have demonstrated significant efficacy, although they are modulable and can be further optimized to serve different purposes. The NPs or NL-NEs have a size of 100–200 nm, polydispersity (PDI) of 0.1–0.2 (which indicates a monodisperse population), and abilities to cross the blood-brain barrier (BBB) at 5%–15% of injected dose and lower lysosomal pH by 0.5–3.0 pH units. *The reported values for BBB penetration are only applicable to PLGA NPs and AcNPs; PaNPs and acidic NL-NEs have not been characterized. PLGA NPs, Poly(lactic-co-glycolic acid) NPs; AcNPs, Acidic NPs; PaNPs, Photo-activated NPs; Acidic NL-NEs, Acidic nucleolipid nanoemulsions; LA, Lactic acid; GA, Glycolic acid; TFSA, Tetrafluorosuccinic acid; SA, Succinic acid. Schematics were created by BioRender and chemical structures were drawn by ChemDraw.

Having established that the maintenance of optimal lysosomal pH is crucial to autophagic and lysosomal functions, different therapeutic strategies have been developed. Compounds that promote autophagy [74] and mitophagy [75] have been described and reviewed. Here, we focus on the development of small molecules and nanomedicine that specifically target and acidify lysosomes (Fig. 3a). Since V-ATPase is an important target for lysosomal acidification, multiple small molecules (e.g., C381 and EN6) have been developed to modulate this enzyme complex and regulate lysosomal pH (Fig. 3b). C381 has been reported to promote lysosomal acidification in human primary dermal fibroblasts and reduce neuroinflammation and gliosis in *PGRN*-knockout mice and MPTP-treated mice [76]. While the precise mechanism of action remains to be investigated, C381 appears to act at least partially through increasing the V-ATPase activity. Importantly, toxicology studies on C381 have shown no major concerns and C381 has been demonstrated to cross the BBB, suggesting its suitability as a potential drug candidate to be advanced towards clinical trials targeting neurodegenerative diseases by restoring lysosomal acidification. EN6 covalently modifies Cys277 of the V1A subunit of V-ATPase and causes its physical and functional decoupling from the Ragulator-Rag GTPase complex. This leads to inhibition of mTORC1, promotion of V-ATPase proton pumping, and improved lysosomal acidification and autophagic function, all of which contribute to increased clearance of TDP-43 aggregates in U2OS osteosarcoma cell line and in mice, although it is unclear whether EN6 is able to cross the BBB [77].

It has also been reported that cAMP (cyclic adenosine monophosphate) and NKH-477 (a soluble adenylyl cyclase activator) promote lysosomal acidification through protein kinase A and pH-dependent translocation of V-ATPase to lysosomal membrane (Fig. 3c) [78–80]. Apart from V-ATPase modulators, there are also small molecules that target lysosomal ion channels to regulate luminal pH (Fig. 3d) [2]. A prominent example is the small molecule SF-22 and its analog, which activate the TRPML1 channel (a member of the TRP channel family) and display an additive effect in promoting lysosomal acidification in combination with phosphatidylinositol-3,5-bisphosphate, which is an endogenous activator [81]. Tetrandrine is another small molecule that inhibits TPC2, thereby acidifying lysosomes and restoring autophagic degradation of pathogenic tau aggregates [20]. Other small molecules such as curcumin analog C1 and PF11 activate transcription factor EB and increase its nuclear translocation, hence promoting lysosome biogenesis and luminal acidification (Fig. 3e) [82]. Furthermore, mTOR inhibitors OSI-027 and PP242 were identified in a fluorescence-based phenotypic screen as lysosome-acidifying hits in differentiated SH-SY5Y neuroblastoma cells, and increased cathepsin D activity and autophagic function (Fig. 3f) [75].

**Fig. 4:**
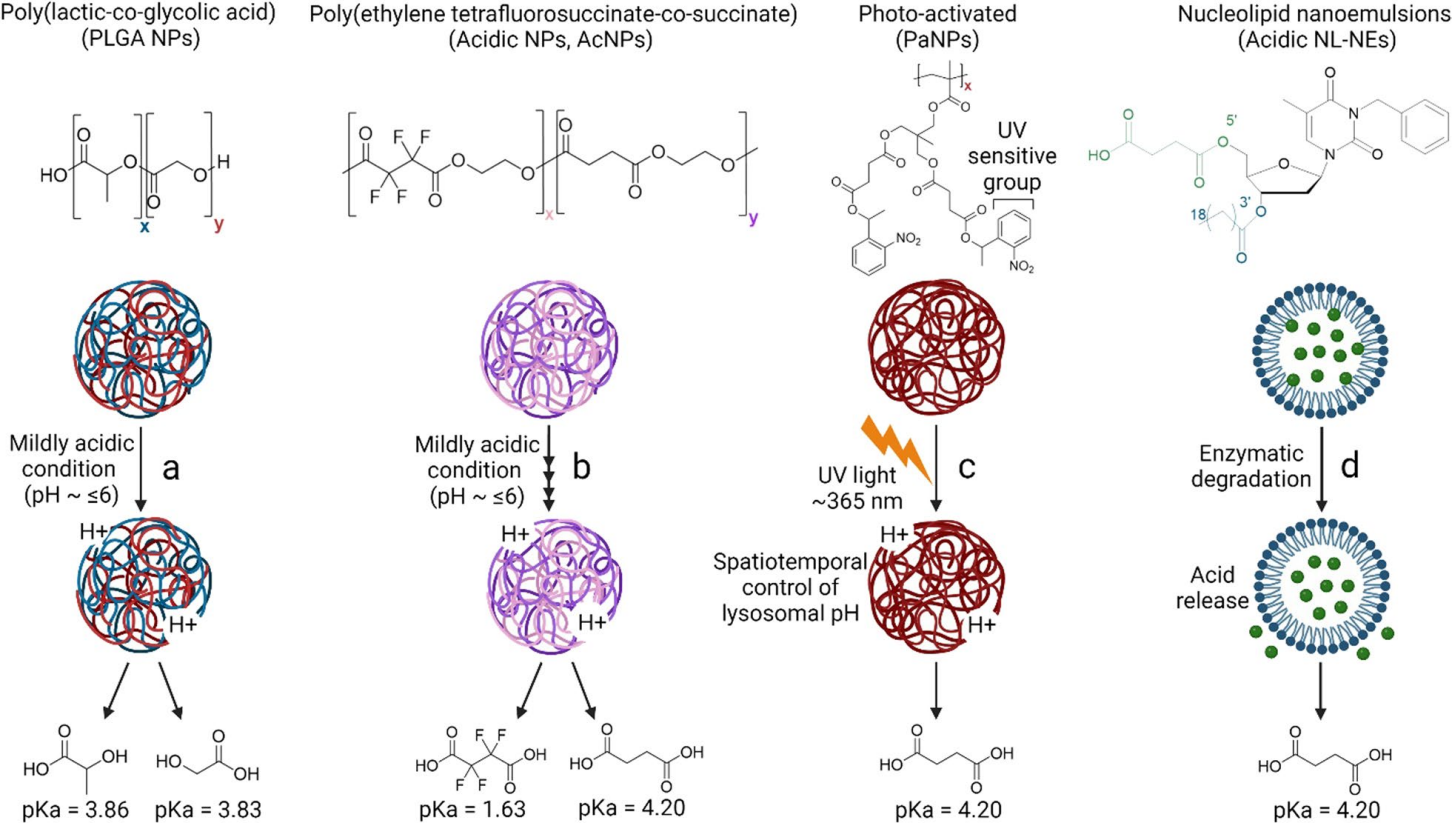
Mechanisms of action of lysosome-targeting nanoparticles (NPs) and nanocarriers. **a** Poly(lactic-co-glycolic acid) (PLGA) NPs will dissociate into their respective component acids, lactic acid (pK_a_ 3.86) and glycolic acid (pK_a_ 3.83), under mildly acidic conditions and directly acidify lysosomes. **b** Acidic NPs (AcNPs) formed by poly(ethylene tetrafluorosuccinate-co-succinate) will dissociate into their respective component acids, tetrafluorosuccinic acid (pK_a_ 1.63) and succinic acid (pK_a_ 4.20), under mildly acidic conditions and directly acidify lysosomes. **c** PaNPs are activated upon stimulation by UV light (~ 365 nm), enabling a spatiotemporal control of lysosomal pH by releasing succinic acid. **d** Acidic nucleolipid nanoemulsions (NL-NEs) are designed to release succinic acid upon enzymatic degradation. Mechanisms of action illustrated are based on the theoretical breakdown of the formed NPs and NL-NEs into their respective component acids. ‘x’ and ‘y’ represent the percentage of each component acid used in the synthesis process. Schematics were created by BioRender and chemical structures were drawn by ChemDraw.

Besides small molecules, there has been constant therapeutic development focusing on using lysosome-targeting nanomedicine, including poly(lactic-co-glycolic acid) (PLGA) nanoparticles (NPs), acidic NPs (AcNPs), photo-activated NPs (PaNPs), and acidic nucleolipid nanoemulsions (NL-NEs), to re-acidify impaired lysosomes under pathological conditions (Fig. 3g) [83–85]. Contrary to small molecules that target specific lysosomal proton pumps and ion channels, these NPs and NL-NEs localize into lysosomes, release their component acids, and directly acidify lysosomes by lowering the luminal pH (Fig. 4) [30, 86]. The NPs are stimuli-responsive; they respond to mildly acidic environments (~ pH 6.0 for PLGA NPs and AcNPs) or ultraviolet (UV) light (~ 365 nm for PaNPs) [30, 32, 86–89]. Notably, PLGA NPs and AcNPs have demonstrated restoration of lysosomal acidification and autophagic function as well as rescue of neurodegenerative pathology in several cellular and mouse models of AD and PD [11, 13, 30, 32, 87, 90], and in a *Drosophila* model of ALS (Fig. 4a, b) [91]. Importantly, AcNPs contain the tetrafluorosuccinic acid with a much lower pK_a_ (1.63) than lactic acid (3.86), glycolic acid (3.83), and succinic acid (4.20), leading to a more efficient increase in lysosomal proton concentration and a higher degree of acidification [87]. On the other hand, PLGA material has been approved by the U.S. Food and Drug Administration as injectable depot formulations such as microparticle [92], although PLGA-based NPs have not been approved for usage and the regulatory hurdles remain to be crossed. PaNPs contain a UV-sensitive nitrobenzyl group that responds to UV light, which enables spatiotemporal control of its activation to release component acid for lysosomal acidification (Fig. 4c), although the requirement for UV stimulation limits their applicability in vivo. An alternative strategy to NPs is acidic NL-NEs that contain conjugated succinic acid which will be released via enzymatic cleavage for luminal acidification (Fig. 4d) [84, 93].

In terms of the pharmacological properties, biodistribution studies have reported that only 5%–15% of the injected dose of lysosome-targeting NPs accumulate in the mouse brain [94, 95]. Due to this limitation, NPs were injected intracranially into the mouse brains in previous studies to ensure direct targeting of CNS cells [30, 32]. It is hence crucial to optimize the chemical formulation or to include BBB-penetrating peptides on the surface of NPs and/or NL-NEs to enable brain targeting [96]. In terms of the functionality of NPs, component acids with lower pK_a_ such as oxalic acid (pK_a_ 1.27) and malonic acid (pK_a_ 2.85) may be incorporated to improve the extent of lysosomal acidification upon uptake and degradation in the lysosomes. However, it is important to optimize the conditions for NP formation and select suitable acids that are well metabolized in the body without inducing side effects. Finally, to translate lysosome-targeting nano-formulations into the clinics, there is a need to ensure batch-to-batch consistency in their size and dispersity, which govern the biodistribution, clearance time, and reproducible scalability, in order to fulfill GMP (good manufacturing practice) quality standards [97].

## Conclusion

In summary, lysosomal acidification impairment and functional defects are integral to the pathogenesis of neurodegenerative diseases. Although studies showed that upregulation of autophagy induction may act as a cellular compensatory mechanism in response to stress under neurodegenerative conditions [98–100], the autophagic degradation will not be completed without fully acidified and functional lysosomes. Numerous studies in genetic and sporadic models of AD and PD have uncovered lysosomal dysfunction to be among the earliest cellular abnormalities and signs of disease pathogenesis before the gradual appearance of histopathological hallmarks such as deposition of toxic protein aggregates and the subsequent synaptic and motor deficits.

Defective lysosomal acidification and function are also prevalent in activated astrocytes and microglia and are associated with impaired phagocytosis and early occurrence of neuroinflammation prior to the initiation of neurodegeneration [101]. This suggests that lysosomal impairment in glial cells exists earlier and contributes to subsequent neuronal dysfunction. In future studies, lysosomal acidification defects in different CNS cell types should be examined by methods including clinical and molecular precision phenotyping [102, 103]. We propose the need to target cellular phenotypes with the earliest signs of impairment.

It is hence imperative to establish tools that enable early detection of lysosomal acidification dysfunction as well as to develop therapeutic agents that can re-acidify impaired lysosomes. There is a need to develop new detection technologies that allow for non-invasive real-time monitoring of lysosomal acidification, especially in humans, for prognosis of neurodegenerative diseases. In terms of therapeutics development, more studies are required to determine the exact mechanisms of action of small molecules and nanomedicine, and their pharmacological properties should be optimized to fit clinical application. These lysosome-targeting detection and therapeutic strategies are generally applicable to other neurodegenerative and neurometabolic disorders as well as demyelinating diseases.

## Data Availability

Not applicable.

## Abbreviations

Aβ: β-amyloid
AcNPs: Acidic nanoparticles
AD: Alzheimer’s disease
ALS: Amyotrophic lateral sclerosis
APP: Amyloid precursor protein
BBB: Blood-brain barrier
CNS: Central nervous system
CSF: Cerebrospinal fluid
FAD: Familial Alzheimer’s disease
FTD: Frontotemporal dementia
IDP: Intrinsically disordered protein
iPSC: Induced pluripotent stem cells
MPTP: 1-Methyl-4-phenyl-1,2,3,6-tetrahydropyridine
NL-NE: Nucleolipid nanoemulsion
NP: Nanoparticle
PaNP: Photo-activated nanoparticle
PD: Parkinson’s disease
PET: Positron emission tomography
PLGA: Poly(lactic-co-glycolic acid)
TMEM175: Transmembrane protein 175
TPC: Two-pore channels
TRP: Transient receptor potential channels
V-ATPase: Vacuolar (H+)-ATPase
